# Mutational profile of skin lesions in hepatocellular carcinoma patients under tyrosine kinase inhibition: a repercussion of a wide-spectrum activity

**DOI:** 10.18632/oncotarget.27891

**Published:** 2021-03-02

**Authors:** Leonardo G. da Fonseca, Carla Fuster-Anglada, Cristina Carrera, Cristina Millán, Esther Samper, Victor Sapena, Álvaro Díaz-González, Marco Sanduzzi-Zamparelli, Cassia Leal, Alejandro Forner, Jordi Bruix, Maria Reig, Loreto Boix, Alba Díaz

**Affiliations:** ^1^Barcelona Clinic Liver Cancer (BCLC) Group, Liver Unit, Hospital Clínic de Barcelona, IDIBAPS, Universitat de Barcelona, Centro de Investigación Biomédica en Red de Enfermedades Hepáticas y Digestivas (CIBEREHD), Barcelona, Spain; ^2^Barcelona Clinic Liver Cancer (BCLC) Group, Department of Pathology, Hospital Clínic de Barcelona, Universitat de Barcelona, Barcelona, Spain; ^3^Melanoma Unit, Dermatology Department, Hospital Clínic de Barcelona, Universitat de Barcelona, Centro de Investigación Biomédica en Red de Enfermedades Raras (CIBERER), Barcelona, Spain; ^*^Authors collaborated equally as first author; ^#^Authors collaborated equally as senior author

**Keywords:** hepatocellular carcinoma, tyrosine kinase inhibitors, treatment adverse events, skin lesions, molecular profiling

## Abstract

Background/Aim: Dermatological adverse events (DAE) in hepatocellular carcinoma (HCC) patients treated with sorafenib predicts better outcome. Some turn into skin lesions (SL) requiring pathology examination. We describe incidence, characteristics and molecular profile of SL in HCC patients treated with sorafenib.

Materials and Methods: SL were prospectively collected in 311 HCC patients who started sorafenib. SL from sorafenib cohort were compared to those from a control patient group selected to match SL type and demographics. *HRAS*, *KRAS* and *BRAF* mutations were analyzed by CAST-PCR, mutated p53 and MAPK pathway activation by immunohistochemistry and immune infiltration by hematoxylin-eosin staining.

Results: Eighty-eight out of 311 patients developed DAE and 7.4% SL required histological assessment. Most frequent lesions were keratoacanthomas (*n* = 4), squamous-cell carcinomas (SCC)(*n* = 5), basal-cell carcinomas (BCC)(*n* = 3) and seborrheic keratosis (*n* = 5). *HRAS* and *KRAS* mutations were detected in 4 SL, while no mutations showed in control SL. Nuclear pERK immunostaining was identified in 33.3% of cases versus 5.3% of controls. Most SL (90%) from patients with DAE were proliferative with intense immune infiltration (73%).

Conclusions: The onset of SL and their molecular profile did not impact negatively on patient’s prognosis, but intense proliferation of SL may reflect compensatory activation of MAPK pathway and warrants their close monitoring.

## INTRODUCTION

Sorafenib is an oral multitarget tyrosine kinase inhibitor directed to vascular endothelial growth factor receptor (VEGFR)-1, -2, -3, platelet-derived growth factor receptor (PDGFR)- b, c-KIT, RET, FLT-3 and BRAF. This agent demonstrated survival benefit in patients with hepatocellular carcinoma (HCC) [1, 2] and represents one of the first line treatments for HCC patients. Besides HCC, sorafenib is also indicated for advanced renal cell carcinoma [3], locally recurrent or metastatic radioactive iodine-refractory differentiated thyroid carcinoma [4] and refractory desmoid tumors [5].

Sorafenib-related dermatologic adverse events (DAE) affect approximately 40% of the patients and are notable for a wide spectrum of manifestations, including maculopapular rash, hand-foot skin reaction, alopecia, xerosis, generalized exanthema and erythema multiforme-Stevens-Johnson syndrome [3, 6]. The occurrence of DAE within the first 60 days, defined as ‘early DAE’ (eDAE), is associated with favorable outcomes in patients with HCC [7–9].

Since the approval of sorafenib, case reports and small series have described the onset of proliferative and inflammatory skin lesions (SL) in patients undergoing treatment, such as squamous cell carcinoma (SCC), keratoacanthoma (KA), actinic keratosis, cystic folliculitis and basal cell carcinoma (BCC) [10–17]. These lesions were predominantly reported in patients with renal cell carcinoma, while anecdotical cases have been described in HCC [11, 12].

It is hypothesized that proliferative keratinocytic SL, specifically SCC, are generated due to a paradoxical activation of the mitogen-activated protein kinase (MAPK) signaling in the presence of a pre-existing mutation in *RAS* [18]. This molecular background differs from the sporadic cases, in which the pathogenesis in highly influenced by UV-radiation-induced mutations in the tumor-suppressor gene *TP53*, that encodes the protein p53 [19].

In daily practice, the occurrence of SL can impair efficacy and quality of life because it often requires treatment interruptions and excisional procedures, potentially leading to functional and/or esthetical sequelae.

There is lack of detailed information regarding the incidence, spectrum of subtypes and molecular hallmarks of SL in HCC patients treated with sorafenib. In the present study, we aimed to describe clinicopathological and molecular features of SL requiring excisional procedures, in a prospective cohort of patients under sorafenib treatment for HCC.

## RESULTS

### Clinical characteristics and treatment outcomes

Between October 2007 and January 2018, a total of 311 HCC patients started sorafenib treatment. The median age of the entire cohort was 63.7 years-old [IQR: 55.7–70.8], 269 (86.5%) patients were male, 278 (89.4%) had ECOG-PS of 0, 270 (86.8%) had preserved liver function (Child-Pugh A). Nine (2.9%) were previously submitted to liver transplantation. Median follow-up was 12 months [IQR: 6.1–22.5], and the median duration of sorafenib treatment was 6.2 months [IQR: 2.1–12.5]. Grade > II DAEs within the first 60 days of treatment (eDAE) were observed in 88 (28.1%) patients (Table 1).

**Table 1:** Baseline characteristics of all patients (*n* = 311) and those who developed SL (*n* = 23)

Patients, *n*	All cohort	SL cohort
	311	23
Age (Years), median [IQR]	63.69 [55.7–70.8 ]	58 [52–66]
Gender (male), *n* (%)	269 (86.5)	19 (82.6)
ECOG-PS (0/1), *n* (%)	278 (89.4)/33 (10.6)	21 (91.3)/2 (8.7)
Child-Pugh score (A/B), *n* (%)	270 (86.8)^*^/41 (13.2)	21 (91.3)/2 (8.7)
Prior liver transplantation (Yes), *n* (%)	9 (2.9)	3 (13)
BCLC stage (A/B/C), *n* (%)	2 (0.6)/139 (44.7)/170 (54.7)	0/12 (52.2)/11 (47.8)
Etiology, *n* (%)		
HCV	126 (40.5)	14 (60.9)
HBV	21 (6.8)	0
Alcohol	71 (22.8)	2 (8.7)
HCV & Alcohol	37 (11.9)	5 (21.7)
NASH	9 (2.9)	1 (4.3)
HIV-HCV	9 (2.9)	1 (4.3)
co-infection HBV-HCV	5 (1.6)	0
Combination of more than one of last categories	5 (1.6)	0
Others	19 (6.1)	0
Healthy liver	9 (2.9)	0

IQR: Inter Quartile Range; ECOG: Eastern Cooperative Oncology Group; BCLC: Barcelona Clinic Liver Cancer; HCV: Hepatitis C Virus; HBV: Hepatitis B Virus; NASH: Non-Alcoholic SteatoHepatitis; HIV: Human Immunodeficiency Virus. ^*^non-cirrhotic patients (*n* = 11).

From the initial cohort, 23 (7.4%) patients developed 32 SL that were submitted to biopsy or excisional procedure, with pathologic assessment. Five patients developed more than 1 SL. The median age of the group who developed SL was 58 years-old [IQR: 52–66], 19 (82.6%) patients were male, 21 (91.3%) had ECOG-PS 0, 21 (91.3%) were Child-Pugh A and 3 (13%) had previous liver transplantation. Eleven (47.8%) were BCLC-C and 12 (52.2%) were BCLC-B. The main liver disease etiologies were chronic hepatitis C virus infection (*n* = 20; 86.9%), alcohol (*n* = 2; 8.7%) and non-alcoholic steatohepatitis (*n* = 1; 4.3%). Coexistence of alcohol and chronic hepatitis C was found in 5 (21.7%) patients (Table 1). The median time from sorafenib initiation to SL biopsy was 9.9 months [IQR: 4.7 to 20.2] and no SL was diagnosed within 60 days of treatment initiation. No differences were found in median time from treatment starting to SL biopsy in eDAE group 10.6 months [IQR: 4.1 to 22.8] and non-eDAE group 9.9 months [IQR: 4.8 to 18].

**Figure 1:**
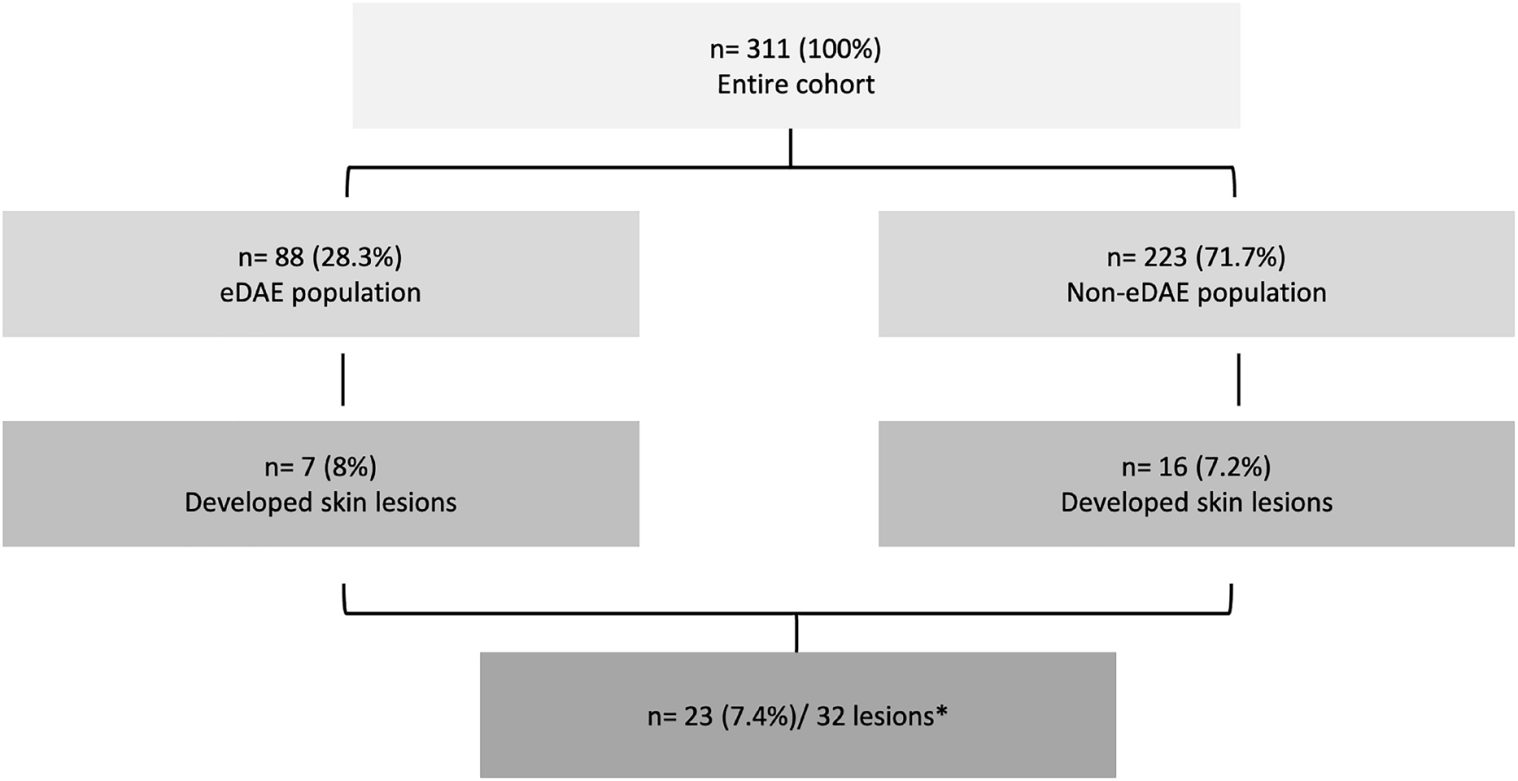
Consort diagram of the patients evaluated in the study. eDAE: dermatologic adverse event within the first 60 days of treatment. ^*^5 patients presented more than 1 skin lesion.

The incidence of SL was similar between the 88 patients who presented eDAE (*n* = 7; 8%) and the 223 patients who did not present eDAE, 16 (7.2%) (Figure 1).

### Clinical outcomes and treatment course

None of the patients required definitive discontinuation of sorafenib due to SL management. The median overall survival of the patients with SL (*n* = 23) was 26.5 months (95% CI 21.6–44) with a median treatment duration of 15.5 months [IQR: 9.5–23]. Patients who develop eDAE had a median overall survival of 18.2 months (95% CI 13.9–23.6) with median treatment duration of 9 months [IQR: 4.5–14]. Finally, those patients with eDAE and SL (*n* = 7) had a median OS of 26.5 months (95% CI 22–51.6) with a median treatment duration of 16.7 months [IQR: 10–22.6]. Detailed clinical characteristics and management are shown in Supplementary Table 1.

### Pathology characterization of skin lesions

**Figure 2:**
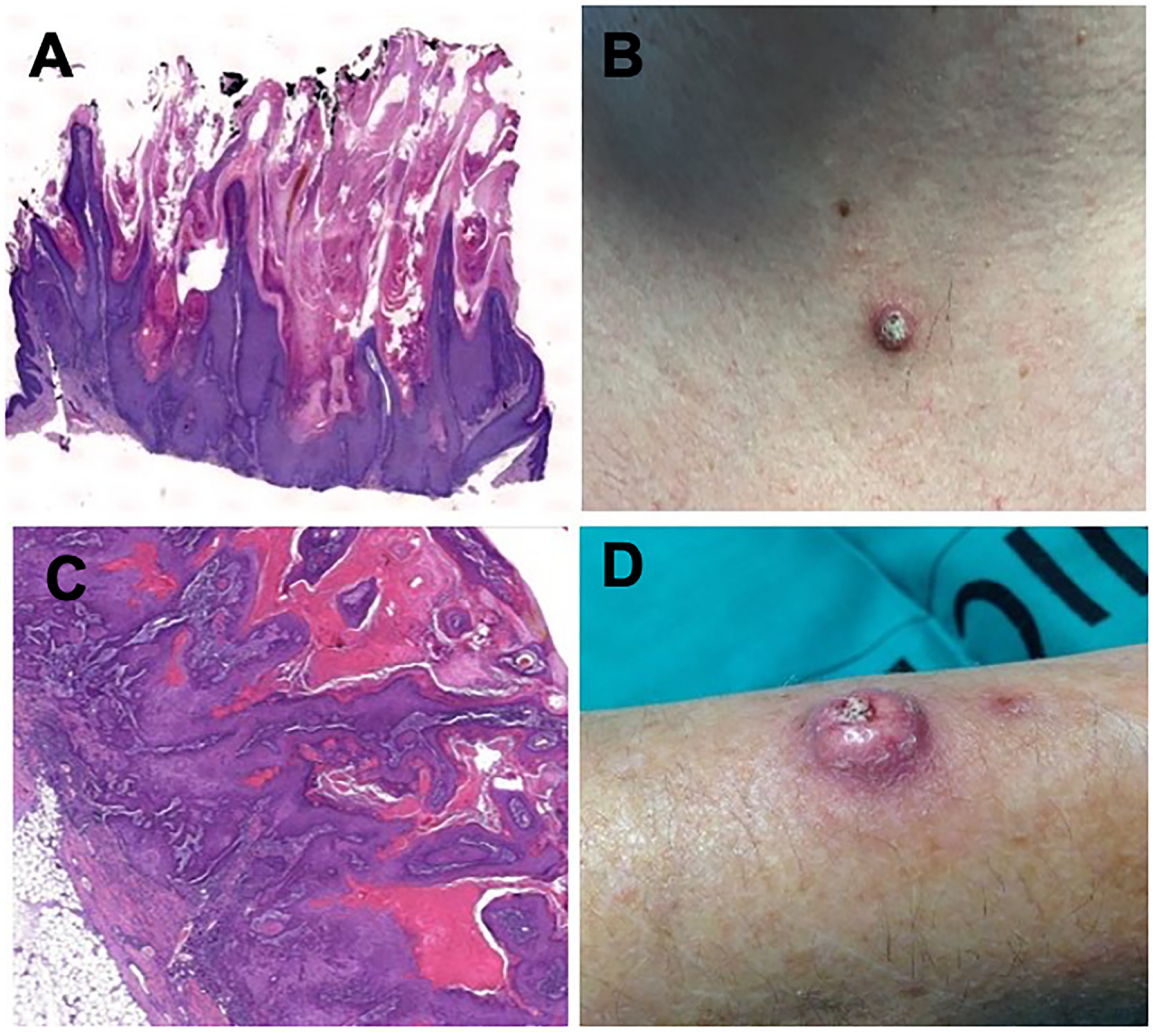
Histologic findings in hematoxylin and eosin staining 2x magnification (**A**) and clinical aspect (**B**) of a keratoacanthoma in a patient under sorafenib treatment. Histologic findings in hematoxylin and eosin staining 2x magnification (**C**) and clinical aspect (**D**) of a squamous-cell carcinoma in a patient under sorafenib treatment.

For additional pathology and molecular characterization, a total of 28 samples of SL from 19 HCC patients were available. We found that 18 of the 28 SL (64.3%) could be classified as proliferative lesions while the rest were non-proliferative entities (*n* = 10/35.7%). The most frequent types of proliferative SL were KAs (*n* = 4/22.2%), SCCs (*n* = 5/27.7%) (Figure 2), BCCs (*n* = 3/6.6%) and seborrheic keratosis (*n* = 5/27.7%). The whole spectrum of SL is depicted in Supplementary Table 2. Marked immune cell infiltration was observed in 15 out of the 18 proliferative lesions (83.3%).

When we divided the 28 SL according to their development in eDAE group of patients (*n* = 11) or non-eDAE patients (*n* = 17) we found that the great majority of SL in eDAE group were proliferative lesions (*n* = 10/ 90.9% vs *n* = 8/47%) with higher immune infiltration (*n* = 8/72.7% vs *n* = 6/35.3%).

### Mutations and immuhistochemistry profile

We further characterized those 28 SL for their *HRAS, KRAS BRAF* and *TP53* mutational status, MAPK pathway activation. These cases were paired with 19 control samples by age, gender and type of SL. The whole description of the mutations found in cases and controls as well as the immunohistochemical findings are described in Supplementary Table 2 and Table 2.

**Table 2:** Clinical and molecular features of control samples

	IHC pERK1/2	IHC P53	HRAS G12D	HRAS Q61K	HRAS Q61L	BRAF V6OOE	KRAS G12D
Squamous cell carcinoma	negative	< 5%	WT	WT	WT	NE	NE
	negative	10%	WT	WT	WT	WT	WT
	negative	30%	WT	WT	WT	WT	WT
Basal cell carcinoma	10%	50%	WT	WT	WT	WT	WT
	negative	20%	WT	WT	WT	WT	WT
	negative	< 5%	WT	WT	WT	WT	WT
	negative	< 5%	WT	WT	WT	WT	WT
	negative	negative	WT	WT	WT	WT	WT
	negative	60%	WT	WT	WT	WT	WT
Seborrheic queratosis	negative	10%	WT	WT	WT	NE	NE
	negative	< 5%	WT	WT	WT	WT	WT
	negative	< 5%	WT	WT	WT	WT	WT
	negative	< 5%	WT	WT	WT	WT	WT
	negative	< 5%	WT	WT	WT	WT	WT
Keratoacanthoma	NE	NE	WT	WT	WT	WT	WT

IHC: immunohistochemistry; pERK: phospho-extracellular signal-regulated kinase; HRAS: Harvey rat sarcoma viral oncogene homolog; KRAS: Kirsten rat sarcoma viral oncogene; BRAF: v-raf murine sarcoma viral oncogene homolog; WT: wild-type; NA: non-available; NE: non-evaluable.

Briefly, we observed a *HRAS* G12D mutation in 1 out of 5 SCC, *KRAS* G12D mutation 1 (out of 3) BBC and in the sebaceous hyperplasia case, *HRAS* Q61L in 1 (out of 3) BCC and a *HRAS* Q61K mutation was found in 1 out of 5 seborrheic keratosis. The latter also presented strong immunostaining for nuclear pERK, suggesting activation of the MAPK pathway. None of the samples presented *BRAF* V600E mutation.

Five out of 24 cases that could be analyzed (20.8%) showed immunostaining for nuclear pERK and only 1 (5.3%) control presented a weak (10%) immunostaining for pERK, suggesting that activation of MAPK pathway was more frequent in HCC patients compared to the control group.

Additionally, we found 4 (16.6%) cases and 8 (42.1%) controls with immune expression of mutated p53 (in > 5% of the cells). This result evidence a higher p53 mutation in control patients compared to sorafenib-treated patients.

## DISCUSSION

We reported the largest series dedicated exclusively to patients with HCC under sorafenib focusing on the onset of SL requiring excisional procedures and their management, mutational profile and clinical outcomes. A wide spectrum of lesions was observed ranging from inflammatory to proliferative profile, with a varying range of time between treatment initiation and biopsy (2.4 to 54.1 months). Despite SL may appear early after sorafenib start, we found no difference in time to biopsy between patients with eDAE and patients without eDAE. Sporadic skin tumors and other proliferative cutaneous lesions development generally occurs with gradual accumulation of genetic mutations along years or decades. The fact that patients receiving sorafenib treatment may develop SL within months after its start, suggest that this agent may hyperactivate the pathways for keratinocyte transformation. While the incidence of SL was similar between patients who presented eDAE (*n* = 7; 8%) and those who did not present eDAE, 16 (7.2%), our study shows that 90% of the SL from patients who developed eDAE, clusters into the proliferative pattern with associated immune cell infiltration in 73% of them. Our study also shows that a more exacerbated and early “skin response” evidenced a longer OS for those patients with eDAE and SL (median 26.5 months 95% CI 22–51.6) compared to that of all eDAE patients (median 18.2 months 95% CI 13.9–23.6) with a higher median treatment time also for eDAE plus SL group. Of note, the longer median sorafenib duration treatment in patients developing SL together with the late onset of SL raise the “chicken or egg paradox” hampering any robust inference in terms of survival.

In order to assess if sorafenib-treated group of patients were enduring a pro-proliferative stimulus for SL development due to a potential paradoxical increase in MAPK signaling pathway in skin cells, we checked mutational status for *BRAF, KRAS, HRAS* genes in our patients compared to mutational status for the same genes in a control group paired according to age, gender, type of skin lesions and demographics. Although none of our samples were positive for *BRAF* V600E mutation, the expression of pERK observed in a number of our samples are in line with the hypothetical paradoxical activation of the MAPK signaling in BRAF wild-type cells already described by other groups [15, 20, 21]. The concept that a targeted agent that blocks a pro-oncogenic mechanism in tumor cell has the potential to activate cell proliferation in a distinct tissue is well-known, not only in patients treated with sorafenib who develop skin cancer, but more frequently with BRAF inhibitors used in melanoma, such as vemurafenib, dabrafenib and encorafenib [22–24]. Once *BRAF* is located downstream from *RAS*, it is proposed that the inhibition of *BRAF* may lead to an upregulation of *RAS*, including *H-RAS*, that is often mutated with keratinocytes with ultraviolet radiation damage, resulting in a *RAS*-induced proliferation and activation of ERK-mediated transcription [20]. In accordance, we did not observe *BRAF* V600E mutations in our cohort. *RAS* mutations were present only in 2 cases of BCC, 1 SCC, 1 seborrheic keratosis and 1 sebaceous hyperplasia. Although the low prevalence of *RAS* mutations both in cases and controls do not fully support the hypothesis of paradoxical activation as it is reported in literature, the hyperactivation of MAPK pathway we have seen in sorafenib-treated group may be due to a compensatory mechanism resulting from its inhibition by sorafenib. Since skin toxicities are frequent in patients receiving multikinase inhibitors, these mechanisms deserves further evaluation in patients under similar agents with kinase inhibition activity.

The current knowledge of skin tumors pathogenesis has been expanding over the last 2 decades [25]. It is reported that 25% of middle-aged people’s skin contains a mutation in one of well-known genes reported to be skin cancer drivers and clones of cells with mutations in p53 gene are reported in the 4% of the epidermis [26]. SCC are molecularly guided by ultraviolet radiation-induced mutagenesis combined with an inability of the immune system to recognize and eliminate mutated cells [27, 28]

The mutation in the gene encoding the tumor-suppressor p53 is a well described alteration in SCC [29], corroborated by the IHC expression both in samples and in controls found in the present study.

Other gene mutations found in SCC include the NOTCH pathway genes, *CDKN2A, HRAS, KNSTRN* and the *KMT2* family [19, 30]. On the other side, KAs origin remains controversial. There are histological similarities between SCC and KA, what could suggest that KA may be part of a spectrum of premalignant lesions associated with SCC. However, the gene expression profile of KA was revealed to be distinct of that of SCC, what suggests that they should be individualized into two distinct entities [31, 32]. The BCC molecular background was clarified by the identification of variants of the *PTCH1*, that is the causative mutation of the Gorlin´s syndrome (an autosomal disorder characterized by early occurrence of multicentric BCC), and by the mechanisms behind the activation of the hedgehog signaling pathways [33]. There are other mechanisms, not explored in the present study, that may also act concomitantly. For example, sorafenib may decrease the transforming growth factor (TGF)-beta by inhibiting the PDGFR. TGF-beta acts on downregulation of cellular proliferation and differentiation, what may turn the keratinocytes more susceptible to oncogenic pathways [24]. Other potential mechanism relies in evidence that sorafenib decrease primary T-cell immune response and impair cutaneous anti-tumor immunosurveillance [21]. Impairment in skin immune environment may be actively involved in the occurrence of inflammatory lesions in our cohort of patients because of sorafenib-treatment and/or immunosuppression regimes after liver transplantation. The association with prior development of DAEs further supports such immune dysregulation. In turn, vascular lesions such as livedo reticularis and thrombotic microangiopathy may derive from the direct impact of sorafenib on endothelial cell regulation and modulation of systemic coagulation in cancer patients [34, 35].

Our study is limited by the low number of patients with each type of SL and to the restricted set of mutations analyzed. This prevents us from drawing a complete causative chain between sorafenib and molecular alterations driving to SL. We expect our data may prime other groups to explore skin lesions under cancer therapy and increase the knowledge in this field. The underlying molecular events and the influence of the immune system should be further explored in order to provide useful information on the impact on these events in the clinical course and outcomes of patients with HCC under any other treatment besides sorafenib. From a clinical point of view, it is important to pay attention to the need to closely monitoring patients in order to detect newly developed SL during treatment.

## MATERIALS AND METHODS

### Patients

A prospective database of consecutive patients treated at Hospital Clinic de Barcelona, from October 2007 to January 2018 was evaluated. Informed consent was obtained from all patients involved in the study that was conducted according to the guidelines of the Declaration of Helsinki and approved by the Institutional Review Board of Hospital Clínic de Barcelona.

Included population consisted of patients with 1) HCC diagnosed based on radiologic and/or histologic features; 2) preserved liver function classified as Child-Pugh class A or B7 (without clinical ascites and/or encephalopathy); 3) Eastern Cooperative Oncology Group performance status (ECOG-PS) of 0 or 1; 4) absence of active cardiovascular disease, except for controlled arterial hypertension and/or stable peripheral vascular disease; 5) adequate renal and hematologic profile, 6) development of a SL detected during sorafenib treatment and 7) requirement of excisional or incisional biopsy for diagnostic and/or therapeutic purposes.

Relevant data were selected including age, gender, ECOG-PS, preexisting liver disease, Child-Pugh score, Barcelona Clinic Liver Cancer (BCLC) stage, prior treatment for HCC, sorafenib treatment duration, toxicities and overall survival. Outcome data were last updated on June 2020.

### Treatment procedures

According to the local protocol, sorafenib was administered orally at starting dose of 400 mg twice daily, which could be modified upon development of adverse events according to its type and severity [36]. The management of DAE and SL was based on a multidisciplinary approach that combined dermatology and hepatology teams. Treatment was continued until symptomatic progression, radiologic progression with option to a second-line trial, treatment intolerance or death. Clinical and laboratory assessments were done monthly while radiology evaluation was done at the end of the first month and bimonthly afterwards. In each clinical evaluation, a complete physical examination including skin inspection was realized by the treating physician. Once a suspected SL was detected, the patient was referred to the dermatology consultant and, if indicated, a biopsy was performed.

### Pathology evaluation and making patients’ study groups

SL samples from patients under sorafenib treatment were collected and sent to Pathology Department. Each skin sample was formalin-fixed, paraffin-embedded and stained with hematoxylin and eosin. SLs were clustered by two pathologists (AD and CF) according to the etiology as 1) proliferative lesions (BCC, KA, SCC and seborrheic keratosis) and 2) non proliferative.

For the control group, samples from patients with SL (6 BCC, 5 KA, 5 seborrheic keratosis and 3 SCC) without diagnosis of HCC nor use of sorafenib were selected. Control group was paired by age, gender and demographics to comparatively characterize molecular and immunohistochemical (IHC) findings.

Immunohistochemistry was used to analyze the nuclear positivity of phospho-ERK 1/2 (pERK) and mutant p53 proteins in SL cells.

Immunostainings were performed on formalin fixed and paraffined tissue sections submitted to antigen retrieval technique using pH 6.0 buffer. Incubation was done with specific antibodies for pERK (Cell Signaling ref 4370 at a 1:300 dilution) and mutant p53 (Abcam ref ab32049 at a dilution 1:400). The result was given as the percentage of SL cells with nuclear expression of pERK and p53 out of the total of SL counted. We considered that nuclear positivity of pERK stands for activation of MAPK pathway. No uniform cut-off has been established in literature for reduced or increased immunostaining of pERK. Based on available evidence, we arbitrarily set the cut-off of 20%, meaning that > 20% of SL cells with positive pERK means activation of MAPK pathway [37]. For p53, a cut-off of > 5% was considered as indicative of p53 mutation [38, 39].

### Mutation detection assay – competitive allele-specificTaqMan-PCR

Mutation analysis was done in the same paraffined-embedded tissues used for IHC analysis. DNA was purified from 10 μm sections using PureLink Genomic DNA mini kit (Invitrogene, ref K1820-02). Detection and measurement of BRAF V600E (BRAF_476_mu: Hs00000111_mu; BRAF_rf: Hs00000172_rf), HRAS G12D (HRAS_484_mu: Hs00000778_mu; HRAS_ref: Hs00001018_rf), HRAS Q61K (HRAS_496_mu: Hs00000785_mu; HRAS_ref: Hs00001018_rf), HRAS Q61L (HRAS_498_mu: Hs00000786_mu; HRAS_ref: Hs00001018_rf) and KRAS G12D (KRAS_521_mu: Hs00000121_mu; KRAS_rf: Hs00000174_rf) somatic mutations was done by means of competitive allele-specific TaqMan™ PCR technology (castPCR™ Technology). The CAST-PCR technology can detect rare amounts of mutated DNA in a sample that contains large amounts of normal, wild-type DNA and works in formalin-fixed paraffin-embedded (FFPE) tissue samples. Real-Time PCR data obtained was analyzed using specific Applied Biosystems™ Mutation Detector™ Software.

### Statistical analysis

Categorical variables were described as absolute frequency and percentages (%). Continuous or ordinal variables were described as median and interquartile ranges [IQR: percentile 25th–75th]. Time to-event data were estimated by Kaplan Meier with median and 95% Confidence Interval (95%CI). All the analysis were performed using SAS v. 9.4 (SAS Institute, Cary, NC).

## SUPPLEMENTARY MATERIALS




