# Supplementary material for: Mutational profile of skin lesions in hepatocellular carcinoma patients under tyrosine kinase inhibition: a repercussion of a wide-spectrum activity
Source: Oncotarget. 2021 Mar 2;12(5):440–9. doi: 10.18632/oncotarget.27891 (PMC7939531; doi:10.18632/oncotarget.27891)
Supplement: Supplementary file 1 [file oncotarget-12-440-s001.pdf]

Mutational profile of skin lesions in hepatocellular carcinoma patients under tyrosine kinase inhibition: a repercussion of a wide-spectrum activity

SUPPLEMENTARY MATERIALS

Supplementary Table 1: Clinical outcomes and treatment course of SL cohort

	BCLC	Events	Patients at risk	Time, months
OS (95% CI), months	All	20	23	26.46 (21.63–43.95)
	B	11	12	25.3 (11.83–43.95)
	C	9	11	41.75 (9.27–51.61)
Follow-up [median IQR], months	All			26.46 [12.62–43.95]
	B			25.3 [16.82–42.82]
	C			26.5 [12.62–49.21]
Time to sorafenib initation to skin lesion [median IQR], months	All			9.93 [4.74–20.19]
	B			13.64 [4.75–22.83]
	C			7.53 [4.44–13.84]
Treatment time [median IQR], months	All			15.45 [9.47–22.98]
	B			16.09 [10.11–21.61]
	C			12.89 [9.04–26.5]

OS: Overall Survival; 95% CI: 95% confidence interval.

Supplementary Table 2: Description of patients who developed skin lesions, regarding baseline features, management and immunohistochemical and mutation profile when available. See Supplementary Table 2
