# Supplementary material for: Mutational profile of skin lesions in hepatocellular carcinoma patients under tyrosine kinase inhibition: a repercussion of a wide-spectrum activity
Source: Oncotarget. 2021 Mar 2;12(5):440–9. doi: 10.18632/oncotarget.27891 (PMC7939531; doi:10.18632/oncotarget.27891)
Supplement: Supplementary file 2 [file oncotarget-12-440-s002.docx]

**Supplementary Table 2**: **Description of patients who developed skin lesions, regarding baseline features, management and immunohistochemical and mutation profile when available**

|  | **Gender, age and etiology** | **Time from sorafenib start to biopsy** | **Type of skin lesion** | **Location** | **Treatment for skin lesion** | **IHC**  **pERK1/2** | **IHC**  **P53** | **HRAS G12D** | **HRAS Q61K** | **HRAS Q61L** | **BRAF V6OOE** | **KRAS G12D** |
| --- | --- | --- | --- | --- | --- | --- | --- | --- | --- | --- | --- | --- |
| **1** | Male, 58 yo HCV- Alcohol. | 8.4 months  11.2 months  11.2 months | - Squamous cell carcinoma - Keratoacanthoma - Keratoacanthoma | Leg  Cheek  Ear | Resection  Resection  Resection | negative  negative  negative | <5%  <5%  <5% | WT  WT  WT | WT  WT  WT | WT  WT  WT | NE  WT  WT | NE  WT  WT |
| **2** | Male, 51 yo HCV-Alcohol | 6.9 months | - Sebaceous hyperplasia | Face | Resection | 50% | <5% | WT | WT | WT | WT | Mutated |
| **3** | Male, 52 yo HCV- Alcohol | 13.1 months | - Keratoacanthoma ^+^ | Neck | Resection | NA | NA | NA | NA | NA | NA | NA |
| **4** | Female, 62 yo HCV | 2.7 months  2.7 months  2.7 months | - Keratoacanthoma - Seborrheic queratosis - Seborrheic queratosis | Scalp  Scalp  Scalp | Resection | NE  negative  negative | NE  <5%  <5% | WT  WT  WT | WT  WT  WT | WT  WT  WT | NE  NE  NE | NE  NE  NE |
| **5** | Male, 56 yo HCV | 9.7 months | - Seborrheic queratosis | Leg | Cryotherapy | 60% | <5% | WT | WT | WT | WT | WT |
| **6** | Male, 64 yo HCV | 54.9 months  54.9 months | - Keratoacanthoma - Squamous cell carcinoma | Elbow | Resection  Resection | 50%  negative | 5%  40% | WT  Mutated | WT  WT | WT  WT | WT  WT | WT  WT |
| **7** | Male, 63 yo HCV, LT | 6.9 months  11.1 months | - Keratoacanthoma - Squamous cell carcinoma^++^ | Arm  Arm | Resection  Resection | negative | negative | WT | WT | WT | WT | WT |
| **8** | Male, 54 yo HIV- HCV | 15.2 months | - Trichilemmal cyst | Face | Resection | NA | NA | NA | NA | NA | NA | NA |
| **9** | Male, 69 yo HCV, LT | 22.0 months  20.4 months  6.9 months | - Basocellular carcinoma - Basocellular carcinoma - Subacute spongiform dermatitis | Back  Back  Thigh | Resection  Resection  Topical bethametasone | negative  negative  negative | negative  negative  10% | WT  WT  WT | WT  WT  WT | WT  WT  WT | WT  WT  WT | WT  Mutated  WT |
| **10** | Male, 67 yo HCV-Alcohol | 10.1 months | - Keratoacanthoma | Ear | Resection | 50% | negative | WT | WT | WT | WT | WT |
| **11** | Male, 73 yo HCV | 4.7 months | - Squamous cell carcinoma | Eyelid | Resection | 30% | 30% | WT | WT | WT | WT | WT |
| **12** | Female, 78 yo HCV | 37.8 months | - Basocellular carcinoma | Leg | Resection | negative | <5% | WT | WT | Mutated | WT | WT |
| **13** | Male, 73 yo NASH | 10.3 months | - Seborrheic queratosis | Hand finger | Resection | negative | negative | WT | Mutated | WT | WT | WT |
| **14** | Male, 65 yo HCV | 41.1 months | - Pilomatrixioma | Elbow | Resection | NA | NA | NA | NA | NA | NA | NA |
| **15** | Male, 59 yo HCV | 6.0 months | - Epi/Hypodermic necrosis | Back | Debridement | negative | negative | WT | WT | WT | WT | WT |
| **16** | Male, 57 yo HCV-Alcohol | 11.5 months | - Suppurative folliculitis | Gluteus | Oral cyprofloxacin | negative | negative | WT | WT | WT | WT | WT |
| **17** | Male, 67 yo Alcohol | 3.1 months  3.1 months | - Livedo reticularis - Livedo reticularis | Legs  Legs | Pentoxifyline | negative | negative | WT | WT | WT | NE | NE |
| **18** | Female 70 yo HCV | 24.6 months | - Interphase dermatitis | Back | Methilprednisolone | NA | NA | NA | NA | NA | NA | NA |
| **19** | Male, 47 yo HCV | 15.2 months | - Septal panniculitis | Leg | Fusidic acid + bethametasone | negative | negative | WT | WT | WT | WT | WT |
| **20** | Female 59 yo HCV | 20.2 months | - Thrombotic vasculopathy ^#^ | Hand finger | Potassium-titanul-phosphate laser | NA | NA | NA | NA | NA | NA | NA |
| **21** | Male, 77 yo HCV | 22.64 months | - Lichen planus ^##^ | Arm | Clobestazole | negative | negative | WT | WT | WT | WT | WT |
| **22** | Male, 73 yo  Alcohol | 2.4 months | - Interphase dermatitis | Trunk | Prednisone | negative | negative | WT | WT | WT | WT | WT |
| **23** | Male, 51 yo HCV | 6.3 months | - Suppurative folliculitis | Back | Unavailable information | NE | NE | WT | WT | WT | WT | WT |

*Yo: years old; HCV: Hepatitis C virus; LT: liver transplantation; HIV: Human immunodeficiency virus; NASH: non-alcoholic steatohepatitis; IHC: immunohistochemistry; pERK: phospho-extracellular signal-regulated kinase; HRAS: Harvey rat sarcoma viral oncogene homolog; KRAS: Kirsten rat sarcoma viral oncogene; BRAF: v-raf murine sarcoma viral oncogene homolog; WT: wild-type; NA: non-available; NE: non-evaluable.*
